# Long sequence single-exposure videography using spatially modulated illumination

**DOI:** 10.1038/s41598-020-75603-7

**Published:** 2020-11-03

**Authors:** Simon Ek, Vassily Kornienko, Elias Kristensson

**Affiliations:** Department of Combustion Physics, Lund University, Lund, Sweden

**Keywords:** Imaging and sensing, Imaging techniques

## Abstract

Frequency recognition algorithm for multiple exposures (FRAME) is a single-exposure imaging technique that can be used for ultrafast videography, achieved through rapid illumination with spatially modulated laser pulses. To date, both the limit in sequence length as well as the relation between sequence length and image quality are unknown for FRAME imaging. Investigating these questions requires a flexible optical arrangement that has the capability of reaching significantly longer image sequences than currently available solutions. In this paper we present a new type of FRAME setup that fulfills this criteria. The setup relies only on (i) a diffractive optical element, (ii) an imaging lens and (iii) a digital micromirror device to generate a modulated pulse train with sequence lengths ranging from 2 to 1024 image frames. To the best of the authors’ knowledge, this is the highest number of temporally resolved frames imaged in a single-exposure.

## Introduction

In a wide range of scientific research fields the ability to record fast transient events in real time is crucial for understanding of the subject at hand, e.g. the delay in photoemission^1^, molecular motions^2^, and photosynthetic systems^3^. High speed cameras have been developed to meet this demand, but due to practical—and eventually even theoretical—limitations in how fast a detector can be read out and made ready to capture the next frame, there is an upper limit on the attainable speed of a high speed camera^4^. To achieve even higher frame rates, an approach that does not rely on fast detectors is needed. One example is the pump-probe methodology, where a transient event is initiated by a pump pulse and probed by another pulse after a controllable time delay. By repeating the process multiple times with varying time delays, the course of the event can be captured with a temporal resolution set by the probe’s pulse length^5,6^. Events that can not be repeated, e.g. due to being stochastic, can, however, not be studied using this method. As a response to this technological gap, a variety of single-exposure techniques, based on illuminating the sample with a train of short laser pulses, have been developed^7^. Since the laser pulses are separated in time, they will each be encoded with image information of temporally distinct parts of the studied event. To retrieve the image information of the individual pulses each pulse must be unique with regard to some characteristic, which can e.g. be angle^8^, spatial position^9,10^ or wavelength^11,12^. Single-shot femtosecond time-resolved optical polarimetry (SS-FTOP)^9^ and light in-flight recording by holography (LIF-DH)^10^ both rely on a spatial separation of the pulses, albeit in different ways. SS-FTOP uses a glass echelon of stepwise increasing thickness, while LIF-DH uses an obliquely sweeping reference pulse to achieve the space division. Compressed ultrafast photography (CUP)^13^ and trillion CUP (T-CUP)^7^ also rely on space division, through the use of a streak camera. However, they are distinct from the other two space division techniques, in that they are indirect imaging techniques, using compressive sensing to reconstruct the sequences. The CUP techniques have recently been further refined into compressed ultrafast spectral photography (CUSP)^14^. Sequentially time all-optical mapping photography (STAMP)^12^ and spectrally filtered STAMP (SF-STAMP)^11^ divert pulses into different parts of the detector, with respect to wavelength. STAMP does so using a transmission grating, while SF-STAMP uses a diffractive optical element (DOE) and a bandpass filter. The above techniques are able to reach picosecond- or, in some cases, femtosecond scale temporal resolution.

In 2017 our research group in Lund developed a new single-exposure filming method, called Frequency Recognition Algorithm for Multiple Exposures (FRAME), which relies on encoding unique spatial modulations into each pulse of the pulse train^15–17^. Although all the pulses reach the same part of the detector, the image information carried by the individual pulses are separated in Fourier space, according to the superimposed modulation of the pulses. This allows the sequence of frames, carried within the pulse train’s constituents, to be reconstructed. Since the size of Fourier space is constant for a given sensor, if more frames are added each frame has to be reconstructed using fewer of the Fourier components, to avoid introducing neighbouring frames (crosstalk) in the reconstructed frame. Therefore the average image quality of the sequence will decrease as the sequence gets longer. As for most, if not all, single-exposure techniques, the trade-off between sequence length and image quality is thus inherent to FRAME. For single-exposure techniques that separate pulses into distinct parts of the detector—space division techniques—a *k* times increase in the number of frames, *n*, decreases the number of pixels per frame, *k* times. For FRAME there is no such simple relation between *n* and pixels per frame. As mentioned, the decrease in the average image quality of the sequence in FRAME is a consequence of having to use fewer Fourier components per reconstructed frame. In practice this means that more high frequency components are excluded as the number of frames increase. This approach is analogous to standard image transform compression, such as JPEG, where removing spatial frequencies with low amplitude—in practice, often the high frequency components—is the means used to achieve compression^18^. Thus far, the low sequence length has thus far been regarded as a limitation of the FRAME concept^7^. In a 2017 FRAME experiment a femtosecond laser system was used to create a video sequence of a light pulse in flight with a temporal resolution of 200 fs^15^. The pulse train was constructed by splitting the output of a single femtosecond laser using beam splitters into four pulses, thus setting the sequence length. However, to be able to investigate whether longer sequences are possible with FRAME, an optical arrangement that does not rely on beam splitters is needed. With beam splitters, the setup would grow in size and complexity with each additional pulse and thus be too bulky and impractical to handle for sequence lengths above $\sim 10$.

Here we demonstrate a new multiplexing optical arrangement for illumination-based FRAME, that allows for videos consisting of up to 1024 image frames: a 250-fold increase compared to what has been previously demonstrated. This is made possible by replacing the traditional beam splitter arrangement with only two optical components; a DOE and a digital micromirror device (DMD). Compared to an optical setup based on beam splitters the current system is both significantly more compact and up to 30000 times more light efficient for longer sequence lengths, which has allowed for the investigation and validation of FRAME’s compatibility with longer image sequences.

## Setup and experimental work

**Figure 1:**
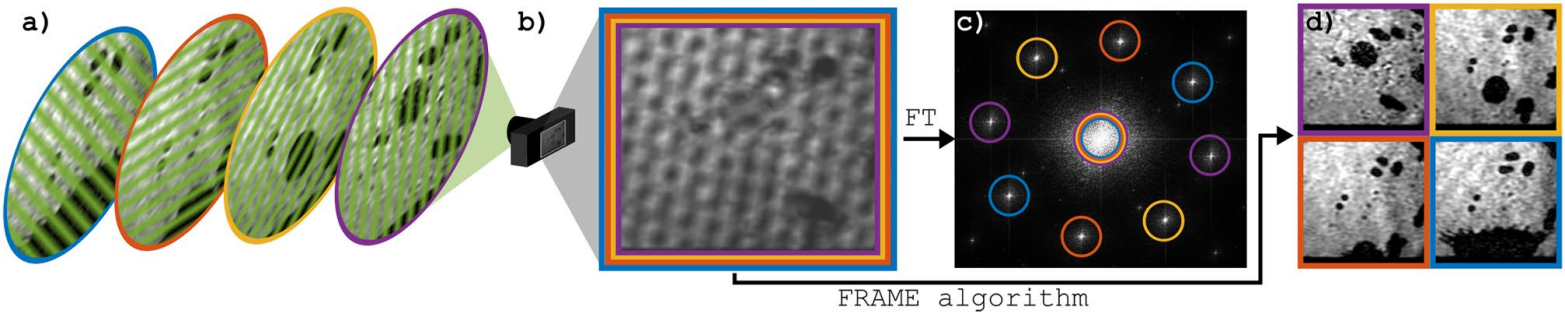
A conceptual illustration of FRAME. (**a**) Four laser pulses, with unique spatial modulations have passed a falling drop. (**b**) The pulses reach the camera and all add up to the detector image. (**c**) The Fourier transform of the detector image reveals the image information being separated according to the spatial modulation of respective pulse. (**d**) By multiplying the image in (**b**) with respective modulation and applying a low-pass filter the individual frames are reconstructed.

FRAME is not one fixed technique, but rather a multitude of experimental methods in combination with a specific post-processing algorithm, used to reconstruct individual frames $F_i$ from an original experimental image *I*^19–22^. Figure 1 shows a flow scheme that explains the principles of FRAME. First of all, the goal of the experimental part is to have a set of light pulses, each with a unique spatial modulation,

$$\begin{aligned} m_i = {cos}(x\nu _{i,x}^\prime + y\nu _{i,y}^\prime + \phi _i), \end{aligned}$$

reaching a detector after interacting with a sample. In the expression, (*x*, *y*) are the spatial coordinates, $\phi _i$ an unknown phase, $\nu _{i,x}^\prime$ and $\nu _{i,y}^\prime$ the components of the spatial frequency $\vec {\nu }_i^\prime$, and *i* an index running from 1 to *n*. Each pulse will be encoded with image information in accordance with the time it interacted with the sample. In Fig. 1a four such intensity-modulated pulses are shown, each carrying information about a falling water droplet at different times. The pulses reach the detector—within a single exposure—and add up to form *I* in Fig. 1b. By Fourier transforming the acquired image (and taking the absolute value of the result), the image in Fig. 1c is obtained. In this domain the majority of the information about the individual frames is confined to small areas, or clusters, each containing the image information of one video frame. In Fig. 1c the first order clusters are separated and can be seen as distinct spots. The central coordinates of these are the frequency components $\pm \vec {\nu }_i$ of the modulations observed by the detector. The *observed* modulations $\pm \vec {\nu }_i$ (in $\hbox {px}^{-1}$) corresponds to the *illumination* modulations $\pm \vec {\nu }_i^\prime$ (in $m^{-1}$). The zeroth order clusters will never be separated, but mixed around the origin in the center. To reconstruct the frame $F_{i=j}$ from *I*, i.e. to go from Fig. 1b–d, involves multiplying *I* by a modulation matrix $M_{\nu _{j}, \theta }$, with frequency $\nu _{j}$ and phase $\theta$, followed by the application of a low-pass filter (LPF). Multiplying *I* with a modulation matrix shifts the entire Fourier domain such that the corresponding frequency component ends up in the origin in the center. A subsequent application of a LPF removes all but the primary frequency components for the frame, resulting in it’s reconstruction. The complete algorithm step to reconstruct the *j*’th frame is:

1 $$\begin{aligned} F_{j} = \sqrt{{LPF}_\sigma (I\odot M_{\nu _{j}, \theta })^2 + {LPF}_\sigma (I\odot M_{\nu _{j}, \theta +\pi /2})^2}, \end{aligned}$$

where $\sigma$ is the full width at half maximum (FWHM) of the LPF. The maximum $\sigma$ that can be used, while still avoiding crosstalk between frames, is dictated by the distance between the spots in Fig. 1c.

For FRAME to work in the context of videography, each light pulse must be temporally controlled and spatially modulated. The modulation can either be achieved by imaging Ronchi gratings with different orientation and/or frequency onto the sample or by the interference of two coherent beams. A benefit with the latter approach is that the intensity modulation naturally forms a pure sine-wave, which will only generate zeroth and first order clusters in Fourier space, whereas Ronchi gratings produce a square wave modulation, also yielding additional higher order cluster, which may interfere in the image post-processing.

**Figure 2:**
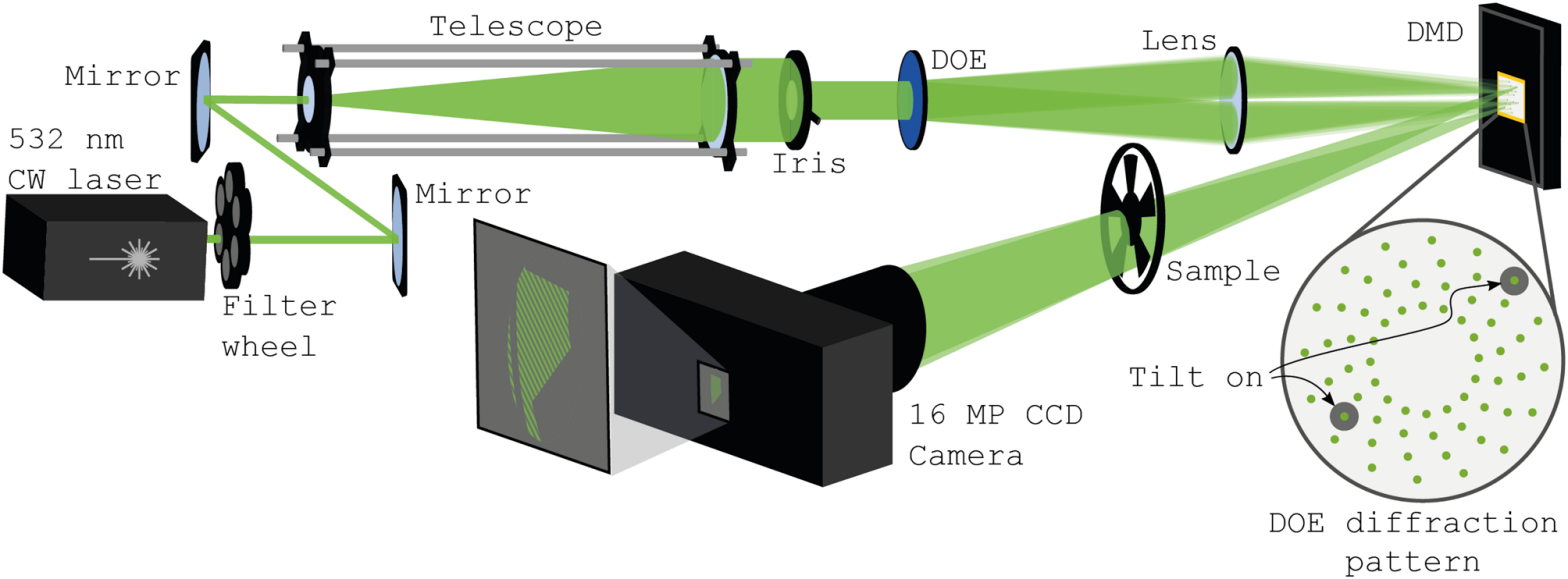
A schematic drawing of the optical arrangement. The DOE and DMD are used to create a train of uniquely modulated pulses, which illuminate the sample. The circular inset shows the DOE diffraction pattern together with a highlighted beam pair, experimentally selected by setting a proper tilt-on pattern on the DMD.

The setup we designed and built to investigate whether FRAME is compatible with long sequences relies on the method of overlapping coherent beams to create an interference pattern. Figure 2 shows a schematic drawing of the setup, which utilizes a continuous 532 nm diode laser (O-Like, 200 mW laser module), a diffractive optical element (DOE) (HOLO/OR), an imaging lens (Thorlabs, LA1979-A) and a digital micro-mirror device (DMD) (Vialux, V-9501 VIS) to create the spatially modulated pulse train. The camera used is a B4822 from Imperx with a 3256 by 4880 pixels ($\approx$ 16 MP) KAI-16070 charge coupled device (CCD) detector with 12 bits of grayscale. The setup also includes a filter wheel to adjust the laser intensity, adjustable mirrors for aligning purposes and a telescope in conjuction with an iris for expansion and cropping of the beam. This way the beam incident on the DOE has the desired diameter and a near top-hat intensity profile. A DOE is a commercially available optical element where a piece of glass has been etched on the micrometer scale in order to control the behaviour of an incident wavefront^23^. This allows for the possibility of tailoring a predetermined diffraction pattern without losing light intensity, allowing for the use of DOEs in many different areas of research such as beamshaping^24^, optical tweezer applications^25^ and micropatterning^26^. The DOE for the current experiments was chosen such that the original $532 \, \hbox {nm}$ beam is split into 64 copies with varying horizontal and vertical angles such that the resulting diffraction pattern has the form of the inset of Fig. 2. This diffraction pattern is then focused onto the DMD by a lens ($f = 200 \, \hbox { mm}$).

The DMD consists of 1920 by 1080 micromirrors that can be tilted to direct the beams towards either the sample (tilt on) or a beam dump (tilt off). If the tilt is in its on state, the beams are directed towards the sample onto which the image of the DOE is formed. By setting a proper pattern of tilt on and tilt off on the DMD, two beams can be made to propagate towards the sample and interfere, thus creating a spatial modulation. Changing the pattern on the DMD so that another pair of beams are allowed to propagate towards the sample changes the spatial modulation of the sample illumination. By repeating this procedure in a rapid succession the desired modulated pulse train is created and the light intensity of each modulated pulse stays at a constant 1/32 of the initial light input (the DOE divides the light into 64 beams of equal intensity). Due to the large number of possible combinations of beam pairs, these need to be chosen strategically in order to maximize the use of Fourier space.

The number of spatial modulations that can be achieved depends on the characteristics of the DOE. Generally, the more beams the DOE splits the original beam into, the more unique beam combinations exist. However, the geometry of the DOE beam pattern also matters. The inset in Fig. 2 shows the DOE pattern that we opted to use. This pattern was chosen with the intent of creating sequences of 32 frames and it consists of 64 beams, arranged in four rings of 16 beams each. However, using 64 beams there are not only 32 possible beam pairs but $$\left( {\begin{array}{c}64\\ 2\end{array}}\right) = 2016$$ possible combinations. For the purpose of counting the number of pairs that yield unique spatial modulations these beam pairs can be divided into two categories. The first category contains the 32 pairs that consist of a beam and its “twin”, i.e. all pairs that are identical under a 180° rotation about the centre. All 32 beam pairs in this category yield unique spatial modulations. The remaining $2016-32=1984$ pairs make up the second category. Here, each combination of two beams has a duplicate (mirrored) pair that will give rise to the exact same interference pattern. Therefore, the beams in the two categories can in total be combined into $1984/2+32 = 1024$ pairs that yield unique interference patterns, setting the maximum sequence length of the setup.

Due to the multitude of available unique modulations, there are many ways to choose *n* beam combinations for sequence lengths of $n<1024$. In general, beam pairs were selected to maximize the distance between frequency components in Fourier space, but some combinations that would end up in particularly ill suited regions of Fourier space were removed, in favour of other combinations. Ill suited regions are, e.g., near the origin in the center where all the zeroth order clusters add up, as well as where information about the target’s stationary parts accumulate. The frames in each sequence were reconstructed using an implementation of Eq. (1), with the filter’s FWHM (σ) being set by the minimum distance between any two spots in Fourier space. A selection of the reconstructed frames are presented in the following section.

## Analysis and results

### Perceived image quality

**Figure 3:**
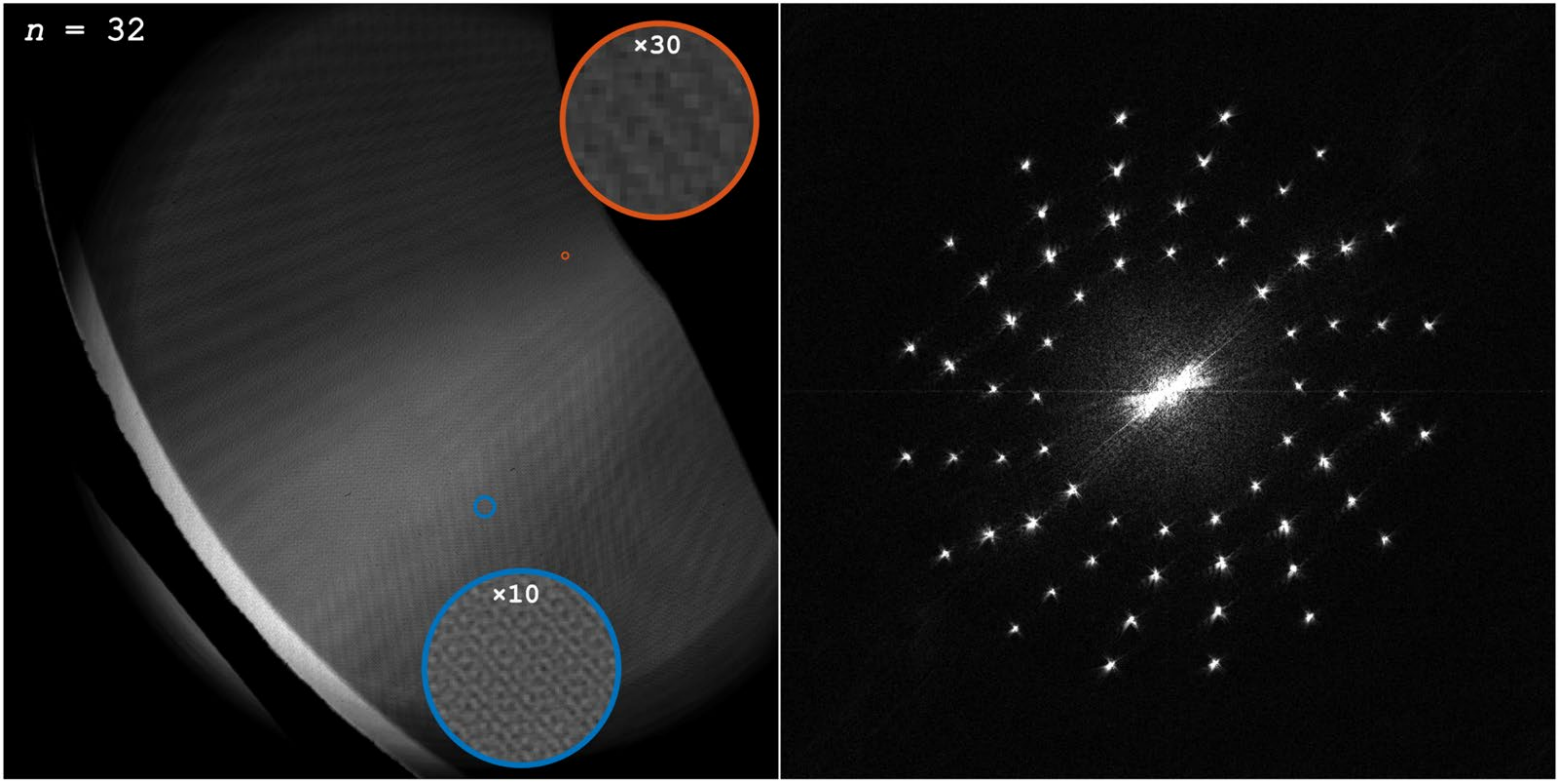
The original detector image of the fan (left) and the absolute value of its Fourier transform (right), when using a sequence length of 32 frames.

**Figure 4:**
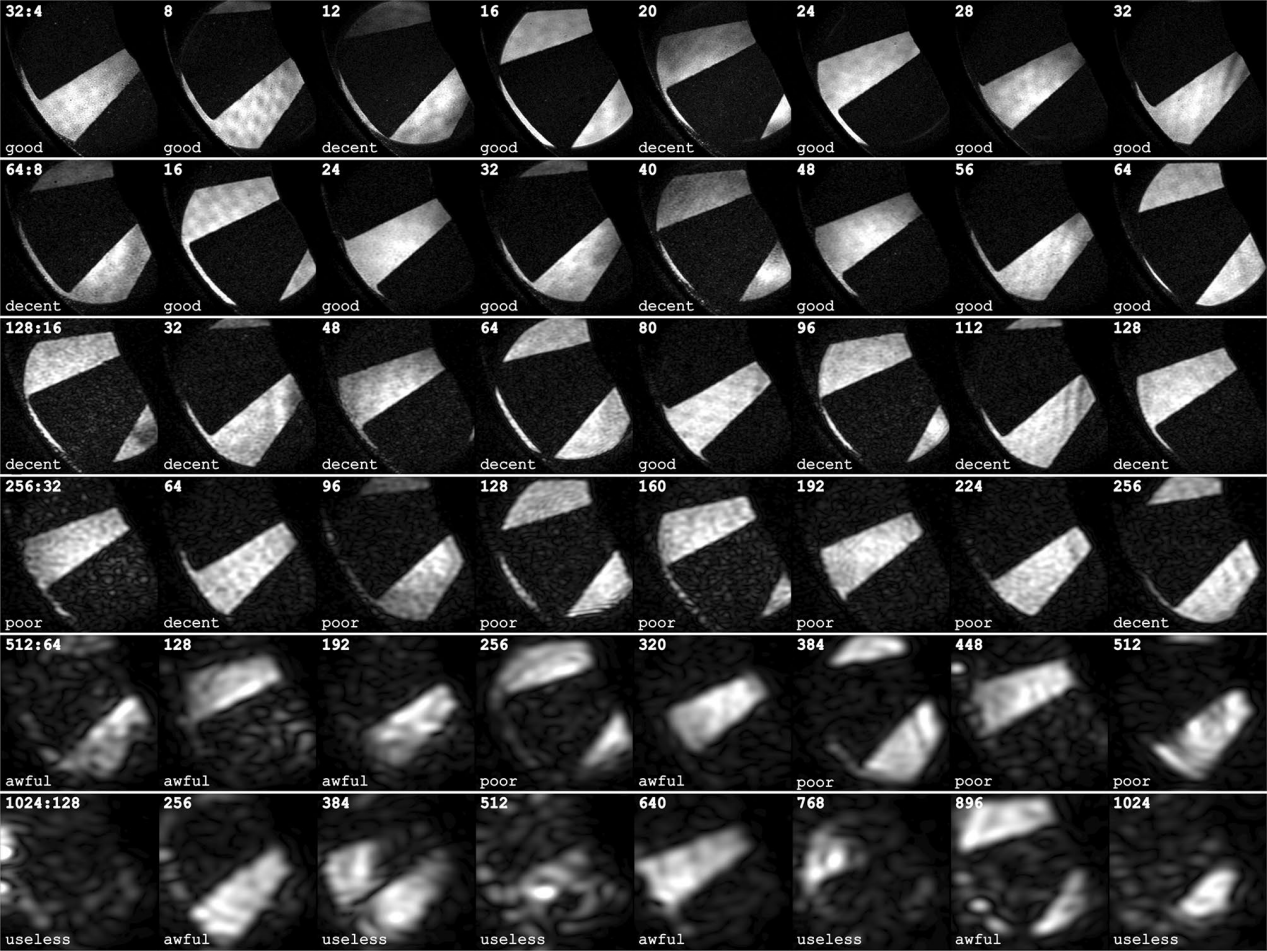
Examples of reconstructed frames for sequences of different length. Each row contains frame number $i \frac{n}{8}$ ($i = 1 \ldots 8$) from a video sequence consisting of *n* frames (indicated for each row). The estimated image quality of each frame is indicated as “good”, “decent”, “poor”, “awful” or “useless”.

In order to investigate how the image quality of the reconstructed frames varies as a function of sequences length, a computer fan, spinning at 3000 RPM, was recorded. Being repeatable, the fan is a suitable sample for this purpose, since essentially the same dynamic event can be filmed multiple times. Also, the low structural complexity of the sample makes it easy to detect flaws and artefacts in the captured frames. The sample was filmed at 10 kfps in sequences consisting of 32, 64, 128, 256, 512, and 1024 frames. The original detector image and its Fourier transform are displayed in Fig. 3 for a sequence of 32 frames. For each sequence eight evenly spaced frames are presented in Fig. 4.

**Figure 5:**
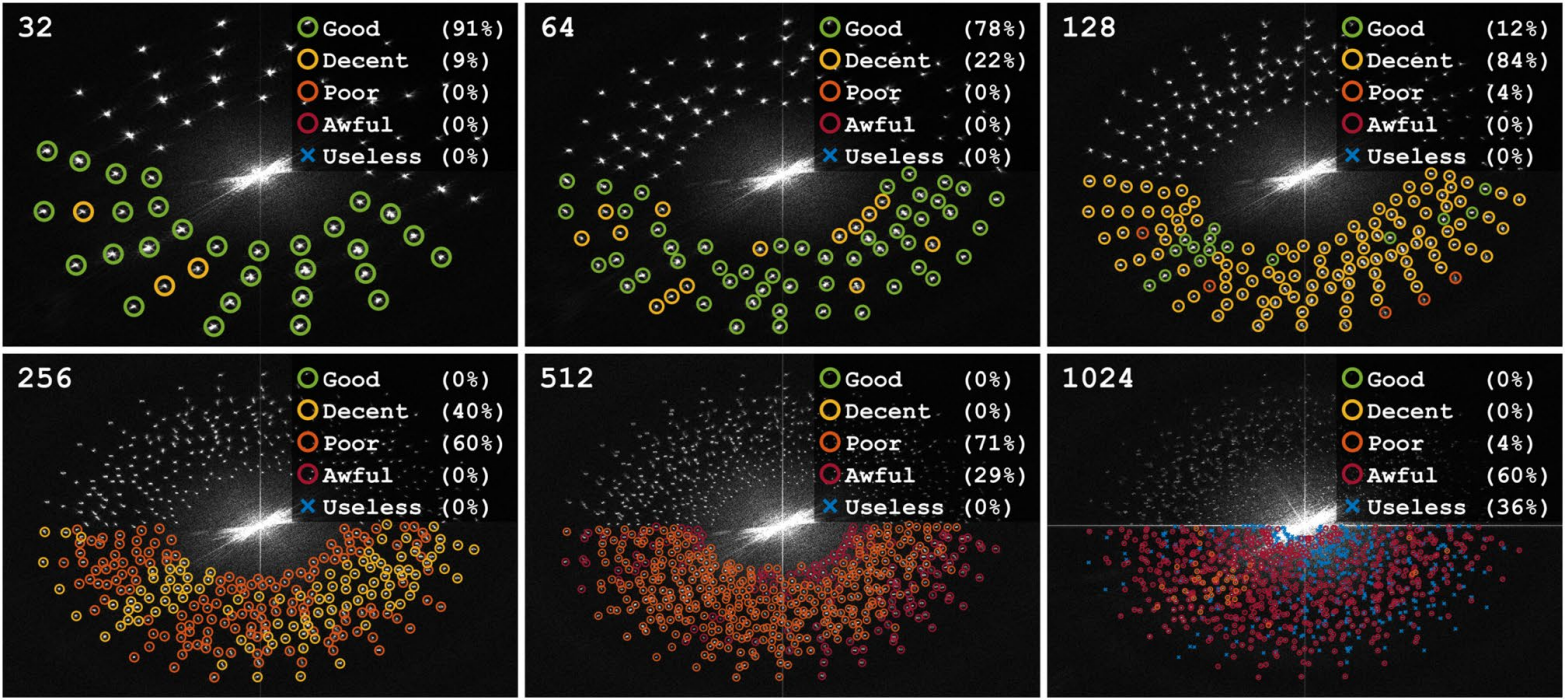
The Fourier transforms of the six original (unprocessed) images, with frequency spots marked according to the image quality of the corresponding reconstructed frame. The images have been cropped to 64% of their original size. The legends show the percentages of each image quality.

In order to make out a trend each frame was attributed, by visual inspection, an image quality on a five-graded scale from “good” to “useless”. Several examples of each quality can be seen in Fig. 4. As expected, due to a decreasing filter size, the trend from this subset is that the image quality gets worse as the number of multiplexed frames, *n*, increases. The more complete and quantitative, but less qualitative, picture of this trend is given in Fig. 5. Here the Fourier transforms of the original images of each sequence are shown, together with spot markings, colored according to the image quality of the corresponding reconstructed frame. The overall trend is a trade-off between sequence length *n* and image quality, which can noted as the quality drops steadily from mostly “good” when $n=32$ to mostly “awful” when $n = 1024$. Figure 5 further shows how the distance between spots decreases with *n* and, consequently, the low-pass filter radii must be reduced with *n* in order to avoid crosstalk between neighbouring frames. At the extreme case where $n=1024$, most of the frequency components are indistinguishable from each other and the spatial resolution of the extracted data is thus very low. However, the results are promising as they show the possibility to encode a significantly higher amount of information into a single photograph using structured illumination than previously achieved and that a more strategic placement of the coded image information could enable better image qualities at long sequences.

### Spatial resolution and data storage capabilities

The FRAME technique exploits the fact that natural images are mostly represented by low spatial frequencies in order to store image data at the vacant high spatial frequencies. FRAME uses intensity modulation to achieve this aim, although similar results can be achieved using e.g. space division methods^7^. As mentioned above, with space division techniques the signals from different time stamps are directed onto different (spatial) regions of the sensor. Each such image frame thus has a reduced number of pixels $N_{\text {frame}}$ that, at best, is equal to the total number of pixels of the sensor *N* divided by the sequence length (number of divisions) *n*, i.e. $N_{\text {frame}} = N/n$. Note that this upper limit of the amount of pixels per image frame is only achieved when the fill factor is 100%, meaning a perfect distribution of the frames across the sensor. Under these ideal conditions, it can be shown that the linear spatial resolution achievable with this approach is reduced by a factor of $1/\sqrt{n}$. To date, the corresponding relationship between image resolution and sequence length for FRAME is not known and requires a more in-depth analysis to be understood. To elucidate, the outcome of the FRAME post-processing of a multiplexed image is a set of image frames, which, in contrast to space division techniques, each have the same number of pixels $N_{\text {frame}}$ as the original (sensor) image, i.e. $N_{\text {frame}} = N$. The entire set of images in the extracted series therefore contains $n \cdot N$ pixels - a factor of *n* greater than the corresponding value for the space division approach. This value, which suggests that FRAME greatly enhances the sensors data storage capabilities, is, however, somewhat misleading since the spatial lock-in algorithm used to extract the data, or more specifically the low-pass filter, leads to a reduced spatial resolution. Instead, each of these oversampled images could be represented by a reduced number of pixels and consequently, a more accurate estimate of the “effective” number of pixels, $N_{\text {eff}}(n)$, in the entire data set is $\alpha (n) \cdot n \cdot N$, where $\alpha (n)$ is the coefficient of oversampling ($0 < \alpha \le 1$). We will now estimate $N_{\text {eff}}$ as a function of *n* in order to investigate FRAME’s data storage capabilities.

**Figure 6:**
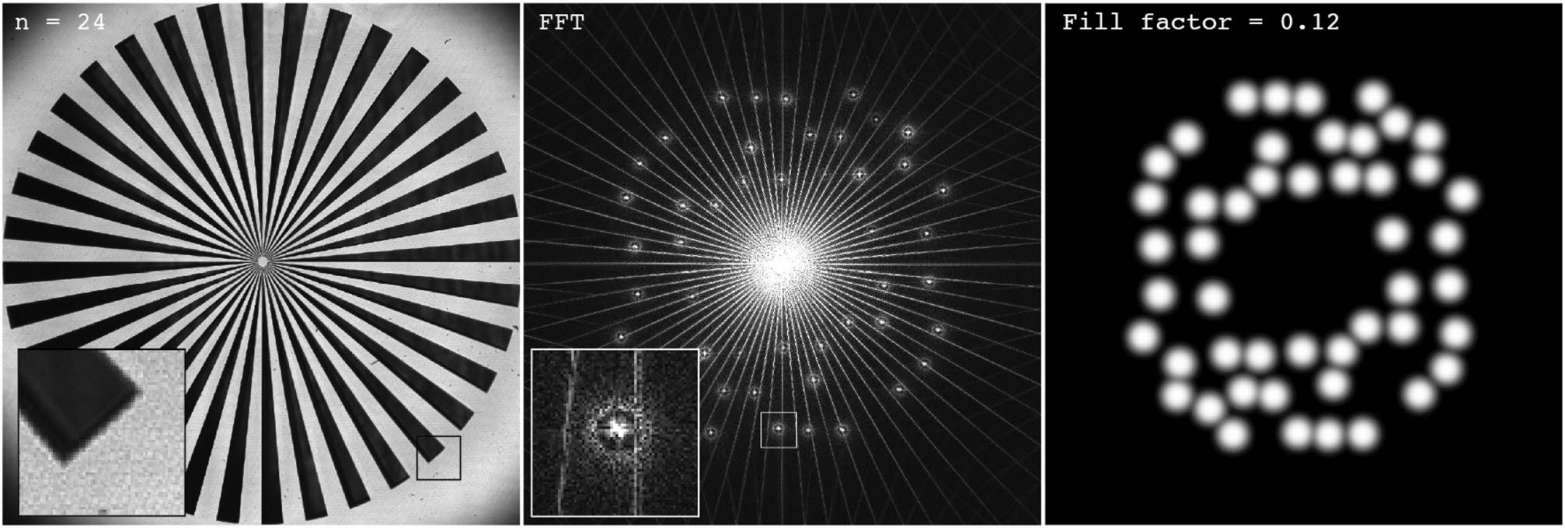
The original detector image of the sector star target (left) and the absolute value of its Fourier transform (middle), when using a sequence length of 24 frames. (Right) Visual representation of all the low-pass filters applied, the sum of which equals the fill factor.

**Figure 7:**
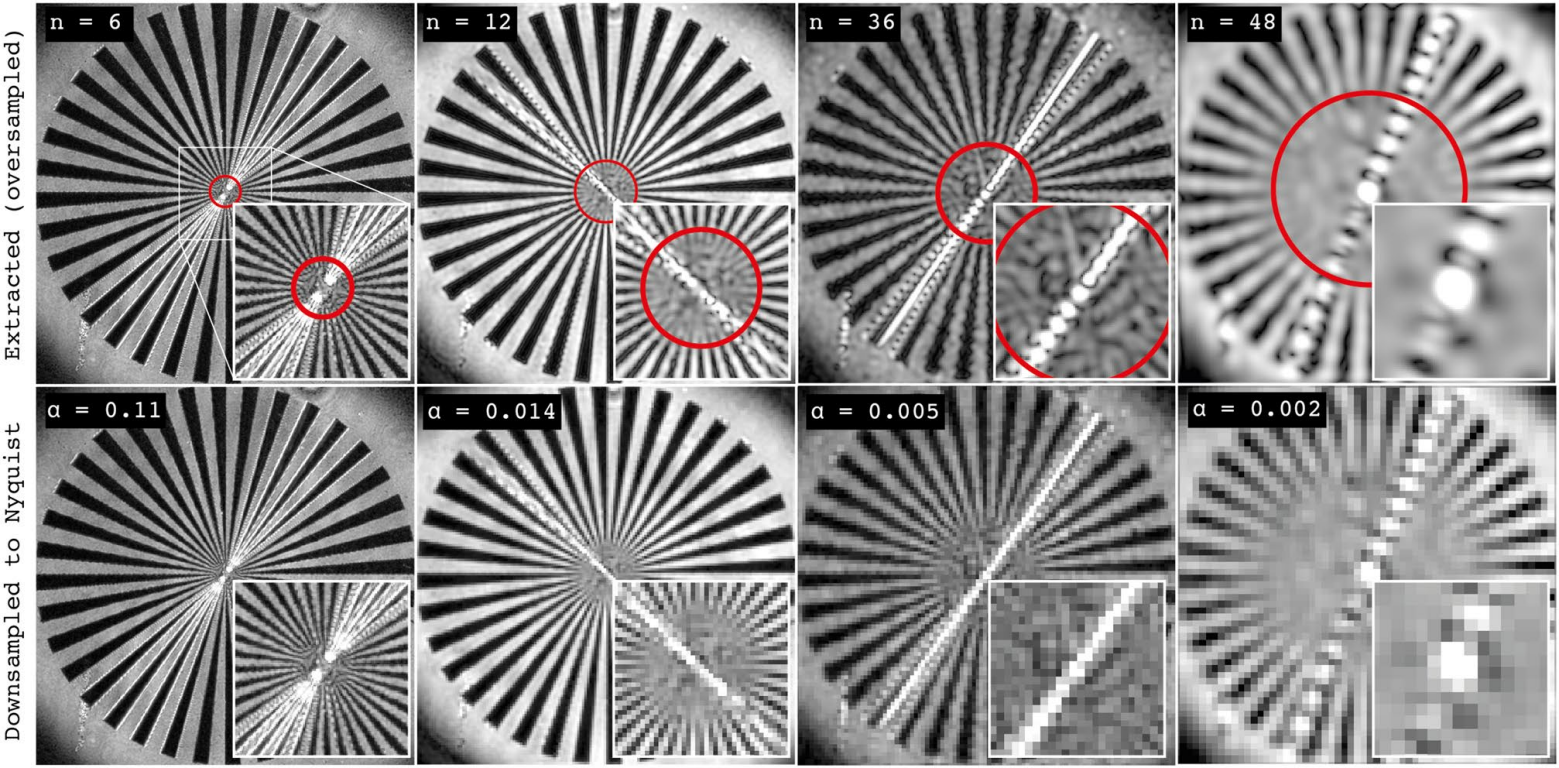
Top row: examples of reconstructed (oversampled) images of the sector star target, showing the location for the 10% contrast cut-off frequency for sequences with 6, 12, 36 and 48 frames. Bottom row: Pixel resolution digitally reduced to match the Nyquist sampling. Note how the image resolution has not changed compared to the top row due to the oversampling nature of the extraction algorithm.

To estimate $\alpha (n)$, a stationary sector star target was filmed at 10 kfps in 15 sequences of different lengths, ranging from $n = 2$ to $n = 256$ frames. The sector star target, which can be seen in Fig. 6, is 10 mm in diameter and consists of 36 black blades with a spatial frequency ranging from 1.15 line pairs per millimeter (lp/mm) at the outer edge to 57.4 lp/mm close to the center. From these measurements, the modulation transfer function (MTF) was extracted and used to find the spatial frequencies at which the reconstructed image frames show 10% contrast between the alternating white and black regions. Note that although the sector star target is a two-dimensional object, the resulting analysis yields a one-dimensional number that represents the overall spatial resolution—in both the x- and y-direction - of the image. This 1D value was then measured for all reconstructed frames in all 15 sequences. The top row in Fig. 7 shows four reconstructed frames from sequences of different lengths together with circles marking the 10% contrast. The general trend can be seen in these images; the longer the sequence, the lower the spatial resolution for the image frames. The sequence with 256 frames was left out of the analysis, since the constituting frames had a 10% cut-off frequency below 1.15 lp/mm (lowest spatial frequency of star target).

**Figure 8:**
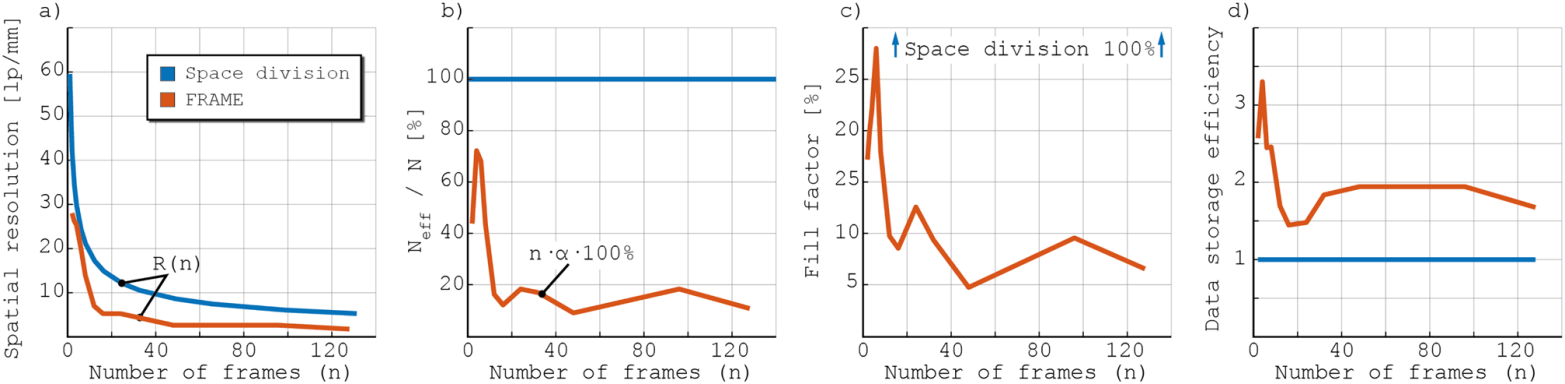
(**a**) 1D spatial resolution, *R*(*n*), as a function of number of frames, *n*, for both FRAME (experimental data) and space division (theoretical data). (**b**) Effective number of pixels, $N_{\text {eff}}$, i.e. the pixels needed to represent the entire data set (video sequence), as a function of *n*. FRAME reaches, at best, 72% of the sensor’s full capacity. (**c**) Fill factor (percentage of the sensor’s number of pixels exploited), as a function of *n*. Here it is assumed that a constant fill factor of 100% can be achieved using space division. By comparing with graph (**b**), one can note that for $n = 3$, 72% of sensor’s full pixel capacity is reached using only a fill factor of 28%. (**d**) The relationship between the trend in (**b**) and that in (**c**), i.e. the data storage efficiency for either technique. While FRAME does not surpass the sensor’s available number of pixels ($N_{\text {eff}}<N$), the curve shows that image data is more efficiently stored in the frequency domain than in the spatial domain.

The MTF analysis of the acquired data shows that the spatial resolution (*R*(*n*)) for the image frames extracted using FRAME reduce more rapidly with sequence length *n* compared to the idealized space division case (Fig. 8a). When combined with the Nyquist theorem, which states that at least two pixels are needed to resolve a single line-pair^27^, these values of 1D spatial resolution can be used to determine the minimum number of pixels, $N_{min}$, needed to display the $10 \times 10 \, \hbox { mm}^2$ camera field-of-view (essentially a conversion into 2D) according to:

2 $$\begin{aligned} N_{min}(n) = (2 \cdot R(n) \cdot 10)^{2}. \end{aligned}$$

The ratio between $N_{min}(n)$ and the sensor’s total number of pixels, *N*, thus equals the coefficient of oversampling α for a given sequence consisting of *n* images:

3 $$\begin{aligned} \alpha (n) = \frac{N_{min}(n)}{N}. \end{aligned}$$

Examples of the extracted data before and after being downsampled are shown in Fig. 7 together with their corresponding $\alpha$ values. The effective number of pixels, $N_{\text {eff}}$, required for the full FRAME video sequence can thereafter be calculated according to:

4 $$\begin{aligned} N_{\text {eff}}(n) = n \cdot N_{min}(n) = n \cdot \alpha \cdot N. \end{aligned}$$

Figure 8b displays $N_{\text {eff}}(n) / N$ in percentage for both space division and FRAME. Unlike the idealized space division case that has a constant $N_{\text {eff}}(n) = N$, FRAME does not exploit all of the sensor’s pixels but reaches a maximum of 72% for $n = 3$ and plateaus at 10–20% for higher *n*. This trend implies that FRAME fails to use the sensor’s full data storage capacity, which is expected as FRAME cannot have a fill factor of 100% (see e.g. Fig. 6). Instead, the fill factor, which for FRAME is the total pixel area covered by the LPFs divided by the total area of the sensor, reach a maximum of 28% at $n=3$, after which it drops to 5–10% at higher *n* (Fig. 8c). The ratio between the effective number of pixels, $N_{\text {eff}}$, and the fill factor indicates the data storage efficiency; for the space division approach, this value cannot exceed unity whereas the corresponding values for the FRAME measurements reach a maximum of about 3.3 (Fig. 8d). This implies that image data is more efficiently stored in the frequency domain than in the spatial domain, or, in other words, that although FRAME makes use of fewer pixels ($N_{\text {eff}}<N$), it exploits them more efficiently. We attribute this trend to the fact that the power of image information is not homogeneously distributed in reciprocal space but primarily concentrated at low spatial frequencies.

### Application on a stochastic target

Unlike pump-probe techniques, FRAME offers the possibility to film transient, unrepeatable events. An example of such a one-time event is the injection of fuel into the cylinders of an engine. To find out whether FRAME can be used to acquire relatively long video sequences of such stochastic and one-time events, a nozzle producing a two spray plumes of water was filmed. The liquid pressure was set to 1.5 bar, making the jets travel at around 17 m/s. To approximately match the duration of the transient event the sequence length was set to 32 frames and the recording speed was set to the highest possible with the setup; 13.3 kfps.

**Figure 9:**
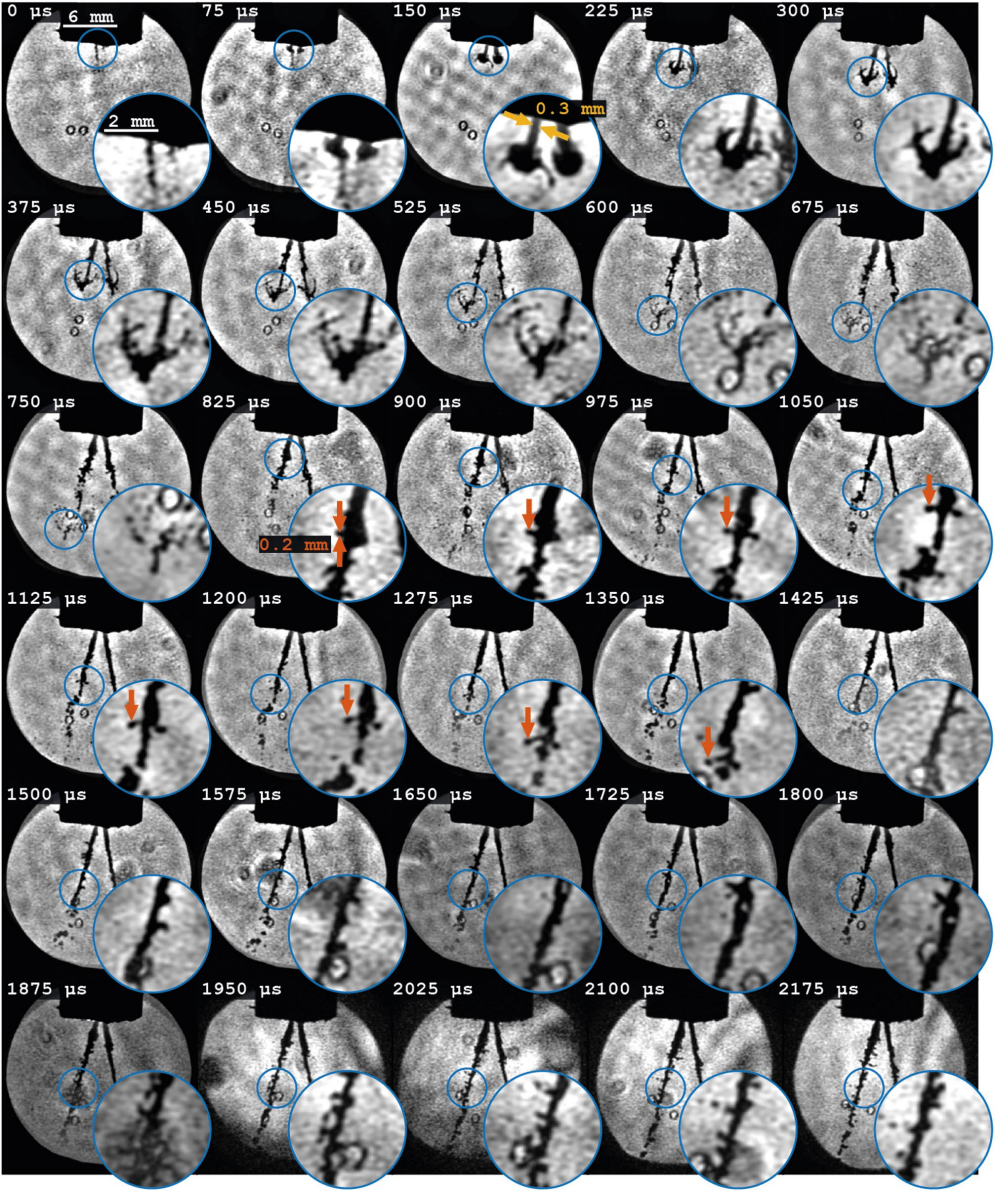
Reconstructed frames from the spray experiment. The insets have been magnified 3 times. The orange arrows indicate a traceable structure of widths of 0.2 mm, i.e. the estimated resolution limit of the system for the a sequence length of $n = 32$, while the yellow arrows indicate the 0.3 mm width of the jet.

The first 30 frames, out of the 32, can be found in Fig. 9, where magnified insets have been added to highlight the ability to capture detailed structural information even at relatively long sequences. For example, the spray plumes, which are approximately 0.3 mm wide, are clearly resolvable, yet finer details can as well be resolved. Theoretically, an object requires a minimum of two pixels (one line pair) to be resolved and therefore structures as small as $1{lp}/R(n=32) \approx 200 {\mu \,m}$ should be detectable, according to the results in section “Spatial resolution and data storage capabilities”. The formation and breakup of such a small liquid structure can be observed and traced over the 8 frames from 825 to 1350 μs.

## Discussion

Though being able to reach extraordinary video recording speeds, FRAME has thus far only been demonstrated with a relatively short sequence length. Here we have demonstrated the compatibility of illumination-based FRAME with long sequence length consisting of up to 1024 individual images: a 250-fold increase from previous demonstrations. This was made possible by greatly reducing the number of essential optical components in the setup to only 3; a DOE, an imaging lens and a DMD. While past solutions to achieve such a long sequence of image frames would have required nearly 2000 beam splitters to form the pulse train, this division is instead accomplished by only *one* DOE. This improvement greatly reduces the physical footprint of the FRAME setup and has a $3 \cdot 10^4$ higher light-division efficiency.

We have demonstrated the applicability of the setup on two dynamic targets; a computer fan and a spray. The known geometry and time evolution of the fan allowed for the attribution of a perceived image quality to each reconstructed frame. Even though the spatial resolution of the individual images decreases as the sequence length increases, the fan blades are clearly traceable for the majority of images even in the 1024 long image sequence (Supplementary Videos 1–6 and Fig. 4). In contrast to the simple geometric structure of the fan, the measurements on the atomizing spray system demonstrated the system’s ability to visualize more complex transient structures and fine image details at sequence lengths up to $n=32$ (Supplementary Video 7).

Due to the fixed etched pattern on the DOE, which was designed for the $n=32$ case, the spread of image information in the Fourier domain could not be optimized for higher *n*, ultimately leading to either cross-talk between the individual image frames or extremely narrow low-pass filters. By redesigning the DOE pattern, the data can be distributed more strategically in reciprocal space, which would boost the fill-factor and thus the overall image quality for $n>32$. The presented results should therefore not be considered as an upper limit for the image quality and sequence length of FRAME but rather as a demonstration of the previously unknown image storage capabilities made possible by the technique.

The development of faster imaging systems is indicative of the growing need to follow physical, chemical and biological processes on time-scales that have not previously been possible. However, the stochastic nature of these processes makes it difficult to synchronize a short acquisition time with the event of interest. Circumvention of this problem necessitates either adjustable acquisition timing or, preferably, longer video sequences. The presented work demonstrates, for the first time, the opportunity of acquiring long video sequences with FRAME for the investigation of such events.

## Supplementary information

Supplementary Legends.

Supplementary Video 1.

Supplementary Video 2.

Supplementary Video 3.

Supplementary Video 4.

Supplementary Video 5.

Supplementary Video 6.

Supplementary Video 7.

## References

[1] Schultze M (2010). Delay in photoemission. Science.

[2] Zewail AH (2000). Femtochemistry: atomic-scale dynamics of the chemical bond. J. Phys. Chem. A.

[3] Berera R, van Grondelle R, Kennis JT (2009). Ultrafast transient absorption spectroscopy: principles and application to photosynthetic systems. Photosynth. Res..

[4] Etoh TG (2017). The theoretical highest frame rate of silicon image sensors. Sensors.

[5] Haight R (1985). Picosecond time-resolved photoemission study of the inp (110) surface. Phys. Rev. Lett..

[6] Downer M, Shank C (1986). Ultrafast heating of silicon on sapphire by femtosecond optical pulses. Phys. Rev. Lett..

[7] Liang J, Wang LV (2018). Single-shot ultrafast optical imaging. Optica.

[8] Li Z (2014). Single-shot tomographic movies of evolving light-velocity objects. Nat. Commun..

[9] Wang X (2014). High-frame-rate observation of single femtosecond laser pulse propagation in fused silica using an echelon and optical polarigraphy technique. Appl. Opt..

[10] Kakue T (2011). Digital light-in-flight recording by holography by use of a femtosecond pulsed laser. IEEE J. Sel. Top. Quant. Electron..

[11] Suzuki T (2017). Single-shot 25-frame burst imaging of ultrafast phase transition of ge2sb2te5 with a sub-picosecond resolution. Appl. Phys. Express.

[12] Nakagawa K (2014). Sequentially timed all-optical mapping photography (stamp). Nat. Photon..

[13] Gao L, Liang J, Li C, Wang LV (2014). Single-shot compressed ultrafast photography at one hundred billion frames per second. Nature.

[14] Wang P, Liang J, Wang LV (2020). Single-shot ultrafast imaging attaining 70 trillion frames per second. Nat. Commun..

[15] Ehn, A. *et al.* Frame: femtosecond videography for atomic and molecular dynamics. *Light Sci. Appl.* **6**, e17045 (2017). 10.1038/lsa.2017.45 PMC6062331 30167293

[16] Kristensson E (2017). Instantaneous 3d imaging of flame species using coded laser illumination. Proc. Combust. Inst..

[17] Dorozynska K, Kristensson E (2017). Implementation of a multiplexed structured illumination method to achieve snapshot multispectral imaging. Opt. Express.

[18] Wallace, G. K. The jpeg still picture compression standard. *IEEE Trans. Consumer Electron.* **38**, xviii–xxxiv (1992).

[19] Gragston M, Smith C, Kartashov D, Shneider MN, Zhang Z (2018). Single-shot nanosecond-resolution multiframe passive imaging by multiplexed structured image capture. Opt. Express.

[20] Dorozynska K, Kornienko V, Aldén M, Kristensson E (2020). A versatile, low-cost, snapshot multidimensional imaging approach based on structured light. Opt. Express.

[21] Li Z (2018). Simultaneous multispectral imaging of flame species using frequency recognition algorithm for multiple exposures (frame). Combust. Flame.

[22] Deng C, Hu X, Zhang JSY, Dai ZZQ (2018). Snapshot hyperspectral imaging via spectral basis multiplexing in fourier domain. Opt. Express.

[23] Wyrowski F (1990). Diffractive optical elements: iterative calculation of quantized, blazed phase structures. J. Opt. Soc. Am. A.

[24] Gu H, Chen M, Wang Q, Tan Q (2018). Design of two-dimensional diffractive optical elements for beam shaping of multicolor light-emitting diodes. Appl. Opt..

[25] Zheng, M. J., Ogura, Y. & Tanida, J. Application of optical tweezers using DOE and SLM to control of beads with information-DNA for photonic DNA computing. In Mu, G., Song, F., Yu, F. T. S. & Jutamulia, S. (eds.) *Inf. Opt. Photon. Technol. II*, vol. 6837, 74 – 82, 10.1117/12.767629. International Society for Optics and Photonics (SPIE, 2008).

[26] Kuroiwa Y, Takeshima N, Narita Y, Tanaka S, Hirao K (2004). Arbitrary micropatterning method in femtosecond laser microprocessing using diffractive optical elements. Opt. Express.

[27] Nyquist H (1928). Certain topics in telegraph transmission theory. Trans. Am. Inst. Electr. Eng..

