# Supplementary material for: Long sequence single-exposure videography using spatially modulated illumination
Source: Sci Rep. 2020 Nov 3;10:18920. doi: 10.1038/s41598-020-75603-7 (PMC7641221; doi:10.1038/s41598-020-75603-7)
Supplement: Supplementary file 1 — Supplementary Legends. [file 41598_2020_75603_MOESM1_ESM.pdf]

Long sequence single-exposure videography using spatially modulated illumination

Simon Ek[1], Vassily Kornienko[1], and Elias Kristensson[1*]

[1]Lund University, Department of Combustion Physics, Lund, Sweden
[*]elias.kristensson@forbrf.lth.se

Supplementary Information

Supplementary Video 1. Full video sequence of the computer fan video in Fig. 4 for $n = 32$.
Supplementary Video 2. Full video sequence of the computer fan video in Fig. 4 for $n = 64$.
Supplementary Video 3. Full video sequence of the computer fan video in Fig. 4 for $n = 128$.
Supplementary Video 4. Full video sequence of the computer fan video in Fig. 4 for $n = 256$.
Supplementary Video 5. Full video sequence of the computer fan video in Fig. 4 for $n = 512$.
Supplementary Video 6. Full video sequence of the computer fan video in Fig. 4 for $n = 1024$.
Supplementary Video 7. Full video sequence of the atomizing spray system in Fig. 9 for $n = 32$.
