# Supplementary figures and images for: Long sequence single-exposure videography using spatially modulated illumination
Source: Sci Rep. 2020 Nov 3;10:18920. doi: 10.1038/s41598-020-75603-7 (PMC7641221; doi:10.1038/s41598-020-75603-7)

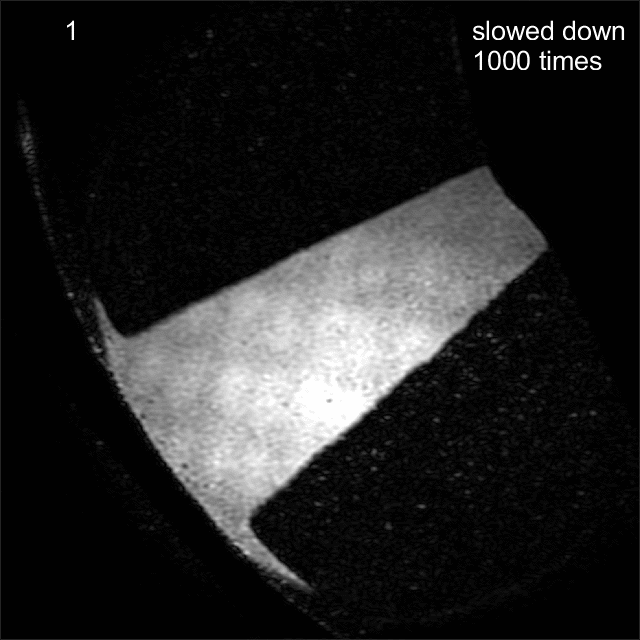

Supplement: Supplementary file 2 — Supplementary Video 1. [file 41598_2020_75603_MOESM2_ESM.gif]

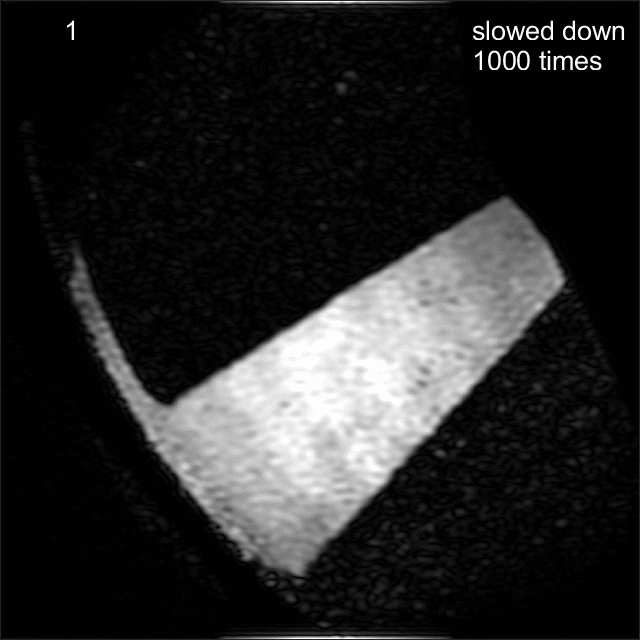

Supplement: Supplementary file 3 — Supplementary Video 2. [file 41598_2020_75603_MOESM3_ESM.gif]

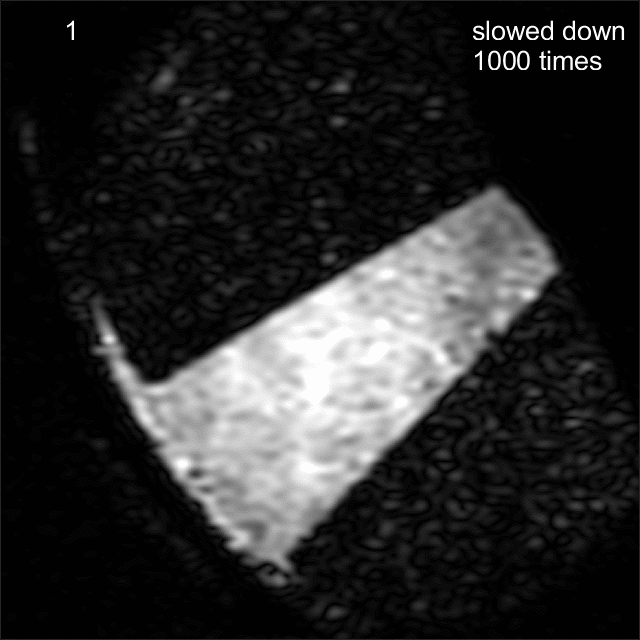

Supplement: Supplementary file 4 — Supplementary Video 3. [file 41598_2020_75603_MOESM4_ESM.gif]

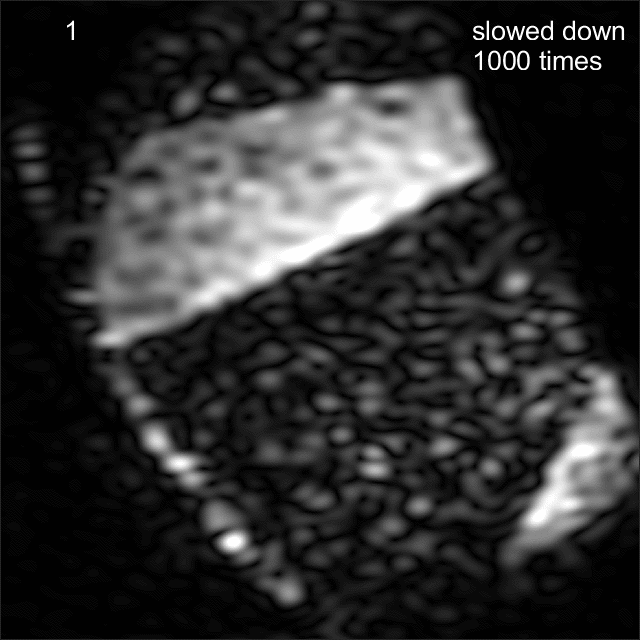

Supplement: Supplementary file 5 — Supplementary Video 4. [file 41598_2020_75603_MOESM5_ESM.gif]

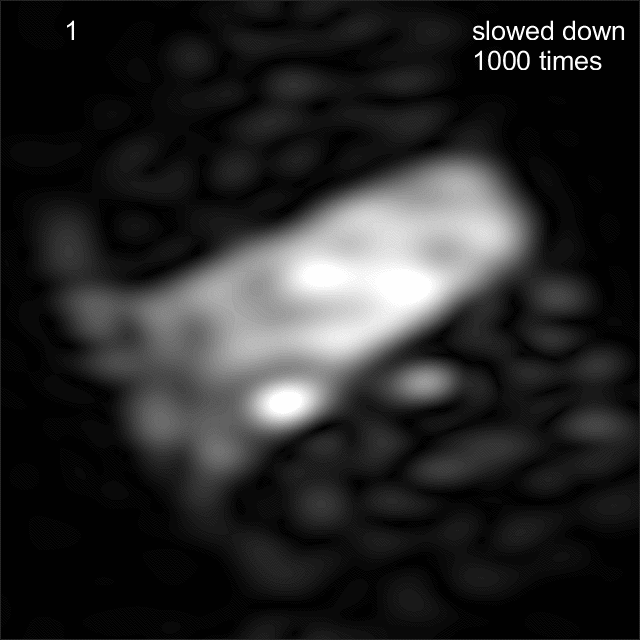

Supplement: Supplementary file 6 — Supplementary Video 5. [file 41598_2020_75603_MOESM6_ESM.gif]

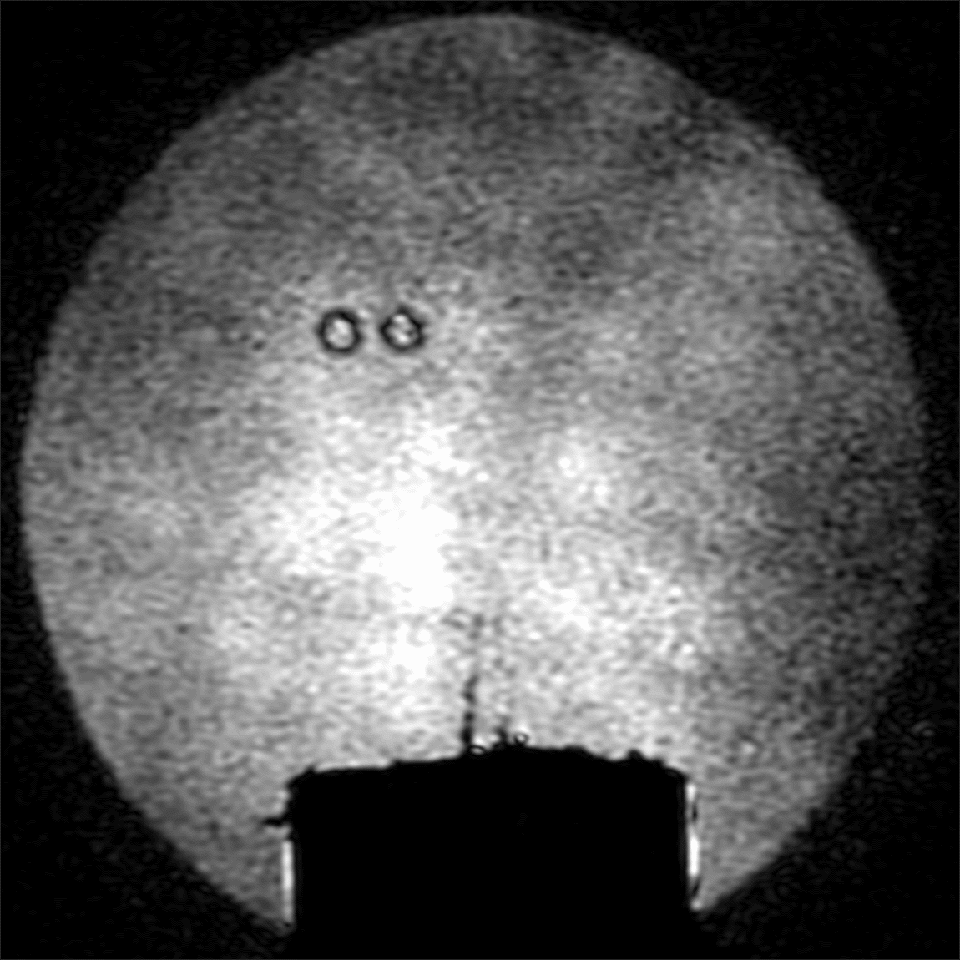

Supplement: Supplementary file 8 — Supplementary Video 7. [file 41598_2020_75603_MOESM8_ESM.gif]
